# Effects of Background Music on Objective and Subjective Performance Measures in an Auditory BCI

**DOI:** 10.3389/fncom.2016.00105

**Published:** 2016-10-13

**Authors:** Sijie Zhou, Brendan Z. Allison, Andrea Kübler, Andrzej Cichocki, Xingyu Wang, Jing Jin

**Affiliations:** ^1^Key Laboratory of Advanced Control and Optimization for Chemical Processes, Ministry of Education, East China University of Science and TechnologyShanghai, China; ^2^Department of Cognitive Science, University of California San DiegoLa Jolla, CA, USA; ^3^Institute of Psychology, University of WürzburgWürzburg, Germany; ^4^Laboratory for Advanced Brain Signal Processing, Brain Science Institute, RIKENWako-shi, Japan; ^5^Skolkovo Institute of Science and TechnologyMoscow, Russia; ^6^Nicolaus Copernicus University (UMK)Torun, Poland

**Keywords:** brain computer interface, event-related potentials, auditory, music background, audio stimulus

## Abstract

Several studies have explored brain computer interface (BCI) systems based on auditory stimuli, which could help patients with visual impairments. Usability and user satisfaction are important considerations in any BCI. Although background music can influence emotion and performance in other task environments, and many users may wish to listen to music while using a BCI, auditory, and other BCIs are typically studied without background music. Some work has explored the possibility of using polyphonic music in auditory BCI systems. However, this approach requires users with good musical skills, and has not been explored in online experiments. Our hypothesis was that an auditory BCI with background music would be preferred by subjects over a similar BCI without background music, without any difference in BCI performance. We introduce a simple paradigm (which does not require musical skill) using percussion instrument sound stimuli and background music, and evaluated it in both offline and online experiments. The result showed that subjects preferred the auditory BCI with background music. Different performance measures did not reveal any significant performance effect when comparing background music vs. no background. Since the addition of background music does not impair BCI performance but is preferred by users, auditory (and perhaps other) BCIs should consider including it. Our study also indicates that auditory BCIs can be effective even if the auditory channel is simultaneously otherwise engaged.

## Introduction

Brain computer interface (BCI) technology has been used to help disabled patients communicate or control external devices through brain activity (Vidal, 1973; Kübler et al., 2001; Blankertz et al., 2010; Zhang et al., 2013a). Noninvasive BCI systems typically rely on the scalp-recorded electroencephalogram (EEG) (Wolpaw et al., 2002; Adeli et al., 2003; Sellers and Donchin, 2006; Allison et al., 2007; Jin et al., 2011a,b, 2012; Ortiz-Rosario and Adeli, 2013; Li et al., 2014; Zhang et al., 2016). Many BCIs require the user to perform specific voluntary tasks to produce distinct EEG patterns, such as paying attention to a visual, tactile, or auditory stimulus (Brouwer and Van Erp, 2010; Höhne et al., 2011; Jin et al., 2011a; Fazel-Rezai et al., 2012; Kaufmann et al., 2014; Cai et al., 2015). We refer to these three approaches as visual, tactile, and auditory BCIs, respectively.

Visual BCIs can yield high classification accuracy and information transfer rate (Kaufmann et al., 2011; Riccio et al., 2012; Jin et al., 2014, 2015; Zhang et al., 2014; Chen et al., 2015; Yin et al., 2016). However, these BCIs are not useful for patients who cannot see. Tactile BCIs have been validated with patients with visual disabilities, including persons with a disorder of consciousness (DOC) (Kaufmann et al., 2014; Edlinger et al., 2015; Li et al., 2016). Devices that can deliver the tactile stimuli used in modern tactile BCIs are less readily available and usable than the tools required for auditory BCIs. Most end users for BCIs do not have vibrotactile stimulators and experience using them, but do have headphones, laptops, cell phones, and/or other devices that can generate auditory stimuli that are adequate for modern auditory BCIs.

Several groups have shown that an auditory P300 BCI could serve as a communication channel for severely paralyzed patients, including persons diagnosed with DOC. Indeed, DOC patients could also benefit from BCI technology to assess cognitive function (Risetti et al., 2013; Lesenfants et al., 2016; Käthner et al., 2013; Edlinger et al., 2015; Ortner et al., accepted). Since many DOC patients cannot see, and have very limited means for communication and control, they have a particular need for improved auditory BCIs.

Auditory BCI systems require users to concentrate on a target sound, such as a tone, chime, or a word (Kübler et al., 2001, 2009; Hill et al., 2004; Vidaurre and Blankertz, 2010; Kaufmann et al., 2013; Treder et al., 2014). Auditory BCIs entail some different challenges from visual BCIs. Compared to vision, sound perception is relatively information-poor (Kang, 2006). Concordantly, the event-related potentials evoked in auditory BCI systems may lead to less effective discrimination between attended and unattended stimuli (Belitski et al., 2011; Chang et al., 2013). To improve the performance of auditory P300 BCI systems, many studies focused on enhancing the difference between attended and ignored events, which could produce more recognizable differences in the P300 and/or other components (Hill et al., 2004; Furdea et al., 2009; Guo et al., 2010; Halder et al., 2010; Nambu et al., 2013; Höhne and Tangermann, 2014). These efforts have made progress, but also show the ongoing challenge of identifying the best conditions for an auditory BCI.

Some work has explored BCIs to control music players and similar systems to improve quality of life. Music could affect users' emotions (Kang, 2006; Lin et al., 2014), which could make BCI users feel comfortable during BCI use. Tseng et al. (2015) developed a system to select music for users based on their mental state (Tseng et al., 2015). Treder et al. (2014) explored a multi-streamed musical oddball paradigm as an approach to BCIs (Treder et al., 2014). Their article presents a sound justification for this paradigm: “In Western societies, the skills involved in music listening and partly, music understanding are typically overlearnt.”

This paper introduces a simple auditory BCI system that includes background music, which we validated through offline and online experiments. This BCI system does not require musical training or expertise. Percussion sounds from cymbals, snare drums, and tom tom drums were used as stimuli and presented over headphones. We chose percussion stimuli because they are easy to recognize, and easy distinguish from each other and background piano music. We hypothesized that subjects would prefer background music, and would not perform worse while background music is playing. Hence, we explored the effect of background music on auditory BCI performance and users' subjective experience, evaluated via surveys. If successful, our approach would render auditory BCIs more ecologically valid.

## Methods and materials

### Subjects and stimuli

Sixteen healthy right handed subjects (8 male, 8 female, aged 21–27 years, mean age 24.8 ± 1.5) participated in this study. Nine of the subjects had prior experience with an auditory BCI. All subjects' native language was Mandarin Chinese. Each subject participated in one session within 1 day. The order of the conditions was counterbalanced across subjects for each session.

All subjects signed a written consent form prior to this experiment and were paid 50 RMB for their participation in each session. The local ethics committee approved the consent form and experimental procedure before any of the subjects participated.

**Figure 1:**
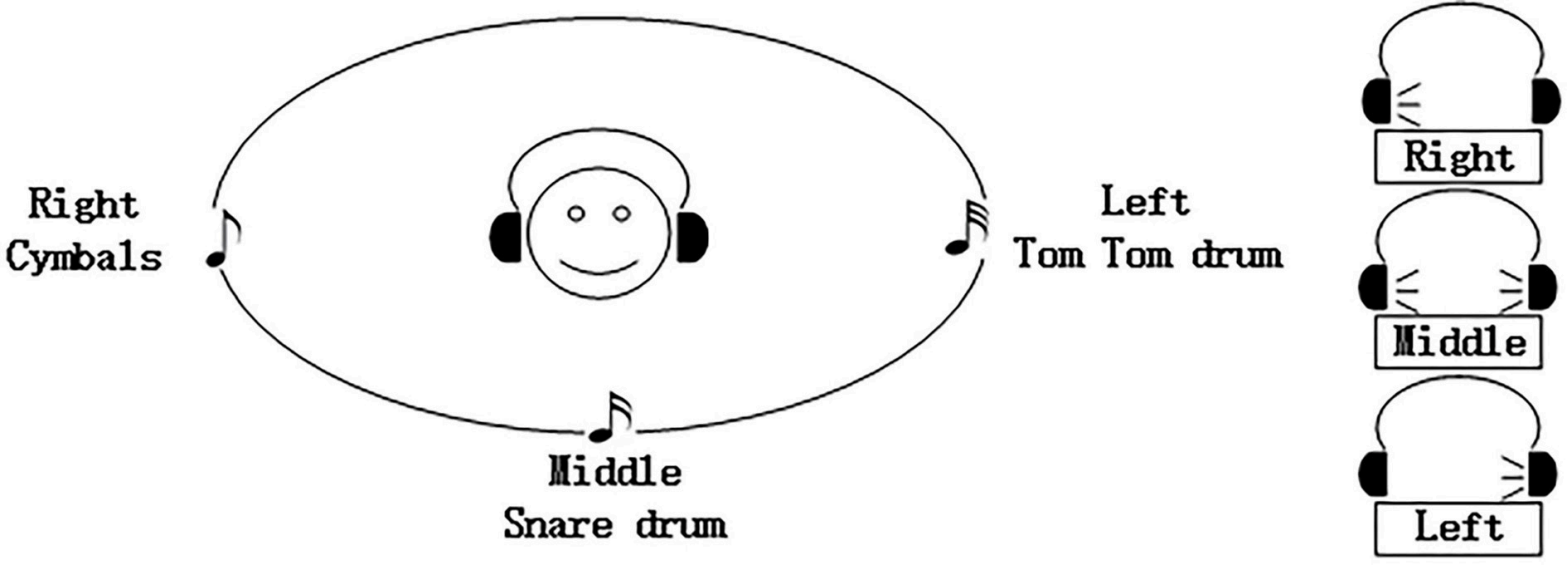
**The three percussion stimuli used in this study, and their spatial distribution**.

Three percussion sounds (cymbals, snare drums, and tom tom drums) were used as stimuli. The “cymbals” stimulus was played in the right headphone, the “snare drum” stimulus was played through both headphones to sound as if it came from the middle, and the “tom tom drum” stimulus was played in the left headphone (see Figure 1). For each subject, we confirmed the subject could hear the stimulus clearly.

### Experimental set up, offline, and online protocols

**Figure 2:**
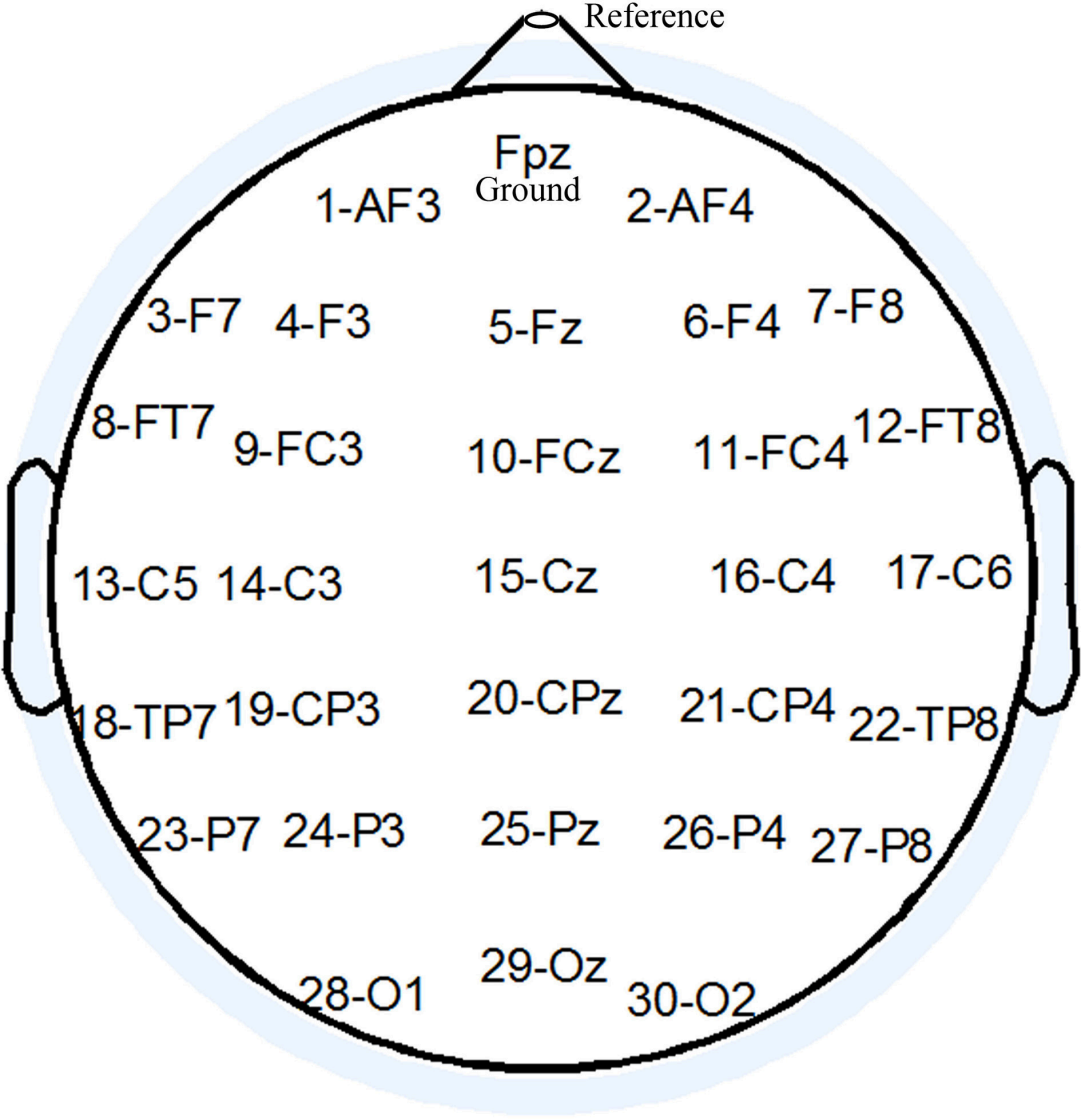
**The electrode montage used in this study**.

EEG signals were recorded with a g.HIamp and a g.EEGcap (Guger Technologies, Graz, Austria) with active electrodes, sampled at 1200 Hz and band pass filtered between 0.1 and 100 Hz. g.HIamp uses wide-range DC-coupled amplifier technology in combination with 24-bit sampling. The result is an input voltage of ±250 mV with a resolution of < 60 nV. The impedance of the electrodes was less than 30 kΩ. Data were recorded and analyzed using the BCI platform software package developed through the East China University of Science and Technology. We recorded from 30 EEG electrode positions based on the extended international 10–20 system (see Figure 2). Active electrodes were referenced to the nose, using a front electrode (FPz) as ground. The recorded data was filtered using a high pass of 0.1 Hz, a low pass of 30 Hz, notch-filtered at 50 Hz for analysis and classification (Käthner et al., 2013). A prestimulus interval of 100 ms was used for baseline correction of single trials.

This study compared two conditions: with no background music (WNB) and with background music (WB). The latter condition used piano music as the background. The piano music was titled “Confession” from Falcom Sound Team jdk. Each subject participated in offline and online sessions for both conditions within the same recording session.

In the offline session, the order of the two conditions was decided pseudorandomly. Each subject completed fifteen runs of one condition, then fifteen runs of the other condition, with a 2 min break after every five runs. Each run contained twelve trials that each consisted of one presentation of each of the three auditory stimuli. At the beginning of each run, an auditory cue in Chinese told the subject which stimulus to count during the upcoming run. The first auditory stimulus began 2 s after the trial began. The stimulus “on” time was 200 ms and the stimulus “off” time was always 100 ms, yielding an SOA of 300 ms. The three auditory stimuli were randomly distributed between stimulus type and corresponding location (see Figure 1), with the constraint that the same stimuli did not occur twice in succession. The target to target interval (TTI) was at least 600 ms. There was a 4 s break at the end of each run, and no feedback was provided. Thus, the offline session took a little over 15 min (0.3 s × three stimuli × twelve trials × fifteen runs × two conditions + a two min break × five times). Subjects had a 5 min break after the offline session.

The online session presented the two conditions in the same order as the offline session. However, there were 24 runs per condition, the number of trials per run was selected adaptively, and subjects received feedback at the end of each run (Jin et al., 2011a). This “adaptive classifier” means that the system would end the run and present feedback if the classifier chose the same output on two consecutive trials. Thus, the minimum number of trials per run was two. Each run still began with an auditory cue (in Chinese) to instruct the subject which target stimulus to count. At the end of the run, the target that the BCI system identified was presented to the subject via a human voice played through the target speaker (left, right, or front), as well as via the monitor. The time required for the online session varied because of the adaptive classifier.

### Classification scheme

The EEG was down-sampled by selecting every 30th sample from the EEG. The first 1000 ms of EEG after each stimulus presentation was used for feature extraction. Spatial-Temporal Discriminant Analysis (STDA) was used for classification (Zhang et al., 2013b). Data acquired offline were used to train the STDA classifier model. This model was then used in the online BCI system. STDA has exhibited superior ERP classification performance relative to competing algorithms (Hoffmann et al., 2008).

### Subjective report

After completing the last run of each session, each subject was asked two questions about each condition. Each question could be answered on a 1–5 rating scale indicating strong disagreement, moderate disagreement, neutrality, moderate agreement, or strong agreement. Subjects were also allowed to answer with intermediate replies (i.e., 1.5, 2.5, 3.5, and 4.5), thus allowing nine possible responses to each question. All questions were asked in Chinese. The two questions were:

1. Did you prefer this condition when you were doing the auditory task?
2. Did this condition make you tired?

### Statistical analysis

Before statistically comparing classification accuracy, the “outputs per minute” and “correct outputs per minute” were statistically tested for normal distribution (One-Sample Kolmogorov Smirnov test) and sphericity (Mauchly's test). Consecutively, repeated measures ANOVAs or *t*-tests with stimulus type as factor were conducted. *Post-hoc* comparison was performed with Tuckey-Kramer tests. The alpha level was adjusted according to Bonferoni-Holm. Non-parametric Kendall tests were computed to statistically compare the questionnaire replies.

## Results

**Figure 3:**
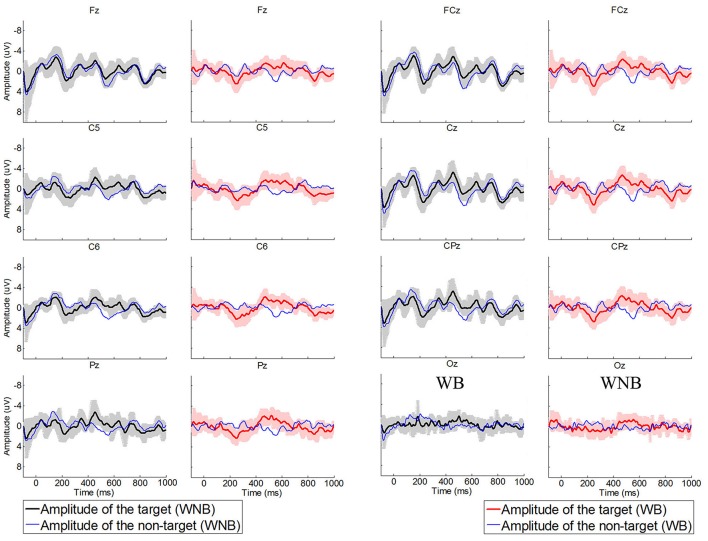
**Averaged evoked potentials of target trials and non-target trials in the high volume session for WNB and WB conditions**.

Figure 3 shows the averaged evoked potentials from the online data over sites Fz, FCz, C5, Cz, C6, CPz, Pz, and Oz. These potentials were averaged from subjects who obtained higher than 70% classification accuracy in all conditions. Figure 3 shows fairly weak negative potentials before 200 ms, and less distinct potentials in occipital areas.

This study had two conditions: WNB and WB. Table 1 shows the online classification accuracy, “outputs per minute,” and “correct outputs per minute” for both conditions. The “outputs per minute” was defined as follows:

(1) $$\begin{array}{r} \text{N}=\frac{60}{\text{Na}\times {\text{t}}_{1}+{\text{t}}_{2}} \end{array}$$

in which N is the “outputs per minute” and Na reflects the averaged trials in a run. The terms *t*_1_ and *t*_2_ denote the time required for a trial and the 4 s break between two runs, respectively.

(2) $$\begin{array}{r} \text{CN}=\text{n}\times \text{acc} \end{array}$$

The CN is the “correct outputs per minute,” and acc is the accuracy of each subject in the online experiment.

**Table 1:** **Online classification accuracy, “outputs per minute” (O/min) and “correct outputs per minute” (CO/min)**.

**Subject**	**Accuracy**		**O/min**		**CO/min**	
	**WNB**	**WB**	**WNB**	**WB**	**WNB**	**WB**
AB38	79.2	100.0	6.4	6.9	5.1	6.9
AB40	95.8	79.2	7.1	6.5	6.8	5.2
AB44	83.3	58.3	6.3	6.8	5.3	4.0
AB49	50.0	58.3	6.7	6.9	3.4	4.0
AB65	58.3	66.7	6.9	6.5	4.0	4.3
AB76	37.5	33.3	6.3	6.1	2.4	2.0
AB77	83.3	83.3	6.6	6.5	5.5	5.4
AB92	83.3	83.3	6.8	7.2	5.6	6.0
AB109	70.8	87.5	6.8	5.8	4.8	5.1
AB112	91.7	100.0	7.6	7.6	7.0	7.6
AB117	83.3	83.3	7.0	6.7	5.8	5.6
AB118	66.7	95.8	6.6	6.5	4.4	6.2
AB119	29.2	50.0	6.5	6.8	1.9	3.4
AB200	87.5	75.0	7.4	6.9	6.5	5.2
AB201	45.8	66.7	6.6	6.2	3.0	4.2
AB202	66.7	66.7	6.1	6.4	4.0	4.2
AVG±STD	69.5 ± 20.2	74.2 ± 18.6	6.7 ± 0.4	6.7 ± 0.4	4.7 ± 1.5	5.0 ± 1.4

Paired samples *t*-tests were used to show the differences between the WNB and WB conditions. There were no significant differences between the WNB and WB conditions in classification accuracy [*t*_(1, 15)_ = −1.2, *p* > 0.05], in “output characters” per minute [*t*_(1, 15)_ = 0.8, *p* > 0.05] and in “correct outputs” per minute [*t*_(1, 15)_ = −0.9, *p* > 0.05]. This result suggests that background music did not affect performace.

Table 2 presents the subjects' replies to questionnaires about the WNB and WB conditions. Non-parametric Kendall tests were used to explore these differences. Results showed a significant preference for background music (*p* < 0.05). Only one subject (AB44) showed a preference for the WNB condition. AB44 also verbally reported that he felt that the background music affected his task performance. There was no significant difference between the WNB and WB conditions in tiredness (*p* > 0.05).

**Table 2:** **Subjective evaluation**.

**Subject**	**Prefer**		**Tiredness**	
	**WNB**	**WB**	**WNB**	**WB**
AB38	2	3	4	3
AB40	3	4	3	3
AB44	4	3	3	4
AB49	3	4	3	3
AB65	3	3	2	2
AB76	3	4	3	3
AB77	3	4	3	3
AB92	3	4	4	3
AB109	4	5	4	5
AB112	3	4	3	3
AB117	3	4	4	3
AB118	3	4	4	3
AB119	3	4	3	3
AB200	4	4	3	3
AB201	3	4	2	2
AB202	1	2	2	1
AVG±STD	3.0 ± 0.7	3.8 ± 0.7	3.1 ± 0.7	2.9 ± 0.9

**Figure 4:**
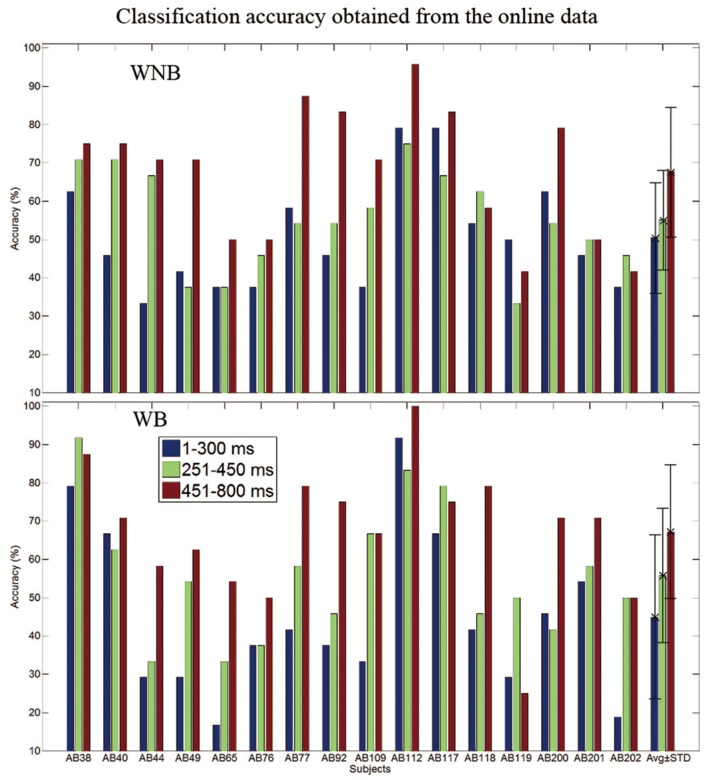
**The contributions of the evoked potentials between 1 and 300 ms, between 251 and 450 ms and between 451 and 800 ms to BCI classification performance, across subjects**.

Figure 4 shows the contributions of ERPs between 1 and 300 ms, between 251 and 450 ms and between 451 and 800 ms for classification performance across subjects. The independent variables were the three time windows, and the dependent variable was the classification accuracy. Figure 4 shows that ERPs between 1 and 300 ms did not contribute strongly to classification, unlike the P300 potential between 250 and 450 ms. Figure 4 also shows that negative ERPs that were predominant between 451 and 800 ms. A two-way repeated measures ANOVA was used to show the classification accuracies based on these time windows [*F*_(2, 30)_, *p* < 0.016]. Potentials between 451 and 800 ms yielded significantly higher classification accuracy than the ERPs between 251 and 450 ms (*p* < 0.016) and the ERPs between 1 and 300 ms (*p* < 0.016), and the potentials between 251 and 450 ms obtained significantly higher classification accuracy compared to the ERPs between 1 and 300 ms (*p* < 0.016). A one-way repeated measures ANOVA was used to test the contributions to classification accuracy among ERPs in different time windows for the WNB condition [*F*_(2, 30)_ = 14.1, *p* < 0.016] and WB condition [*F*_(2, 30)_ = 19.8, *p* < 0.016) respectively. The result showed that the potentials between 451 and 800 ms obtained significantly higher classification accuracy compared to the ERPs between 1 and 300 ms (*p* < 0.016) and the ERPs between 251 and 450 ms (*p* < 0.016), except for the WB pattern. The potentials between 451 and 800 ms did not obtain significantly higher classification accuracy compared to the ERPs between 251 and 450 ms (*p* = 0.029) in WB condition.

## Discussion

### Effects of background music

The main goal of this study was to assess the effects of background music on the performance and user preferences on an auditory BCI. Results showed that the subjects preferred the WB condition over the WNB condition (see Table 2). There were no significant differences in classification accuracy between these two conditions. These results indicate that background music could make auditory BCI users more comfortable without impairing classification accuracy (see Figure 4, Tables 1, 2).

The classification accuracy and information transfer rate in WB condition in the present study were at least comparable to related work. For example, Halder and colleagues presented results with a three stimulus auditory-based BCI (Halder et al., 2010) and discussed training with an auditory-based BCI (Halder et al., 2016). The information transfer rate of the three stimuli BCI was lower than present study (the best of them was 1.7 bit/min). Käthner and colleagues reported that the average accuracy of their auditory-based BCI is only 66% and the SD is 24.8 (Käthner et al., 2013). Some other studies also reported that the average accuracies of their auditory-based BCI were about 70% (Schreuder et al., 2009; Belitski et al., 2011; De Vos et al., 2014). Compared to these studies, the accuracy and information transfer rate in the present study were very common for auditory-based P300 BCI.

### Trial-to-trial interval (TTI) and number of stimuli

This study used a fairly long SOA (300 ms) because our paradigm only used three stimuli. Although we avoided successive repetition of the same stimulus in the same position, shorter TTIs could have made it difficult for subjects to distinguish the different stimuli and reduced P300 amplitude (Gonsalvez and Polich, 2002).

Most P300 BCIs use more than three stimuli. Adding more stimuli and making them more distinct could make a shorter SOA feasible. For example, Höhne and Tangermann (2014) used an 83.3 ms ISI with 26 stimuli, with a duration of 200–250 ms (Höhne and Tangermann, 2014). In their study, the target stimulus was presented after 6–10 non-target stimuli, and the TTI was at least 600 ms, which should be enough for the subjects to detect the target stimulus.

### ERPs and relative contributions to classification accuracy

Figure 4 showed that ERPs before 300 ms contributed to classification accuracy less than ERPs from the other two time windows that we analyzed. Some auditory studies reported that their paradigms could evoke clear mismatch negative potentials (MMNs) (Hill et al., 2004; Kanoh et al., 2008; Brandmeyer et al., 2013). However, there were no clear negative potentials from target trials around 200 ms, due to differences in our stimuli and task instructions. Several factors can affect the MMN, including modality, stimulus parameters, target probability, sequence order, and task instructions (Näätänen et al., 1993; Pincze et al., 2002; Sculthorpe and Campbell, 2011; Kimura, 2012).

Figure 4 shows that the time window between 451 and 800 ms yielded significantly higher classification accuracy than the other time windows (1–300 ms and 251–450 ms) in most comparisons. Thus, late potentials (after 450 ms) contributed to classification accuracy more than early potentials (before 450 ms) in this study.

The evoked potentials were weak in occipital areas (see Figure 3). This result suggests that the BCI presented here might be practical with a reduced electrode montage that does not include occipital sites. Thus, a conventional electrode cap may not be necessary. Alternate means of mounting electrodes on the head (such as headphones) might reduce preparation time and cost while improving comfort and ease-of-use. This could be especially important in long-term use for patients with DOC or other severe movement disabilities, when occipital electrodes can become uncomfortable if the head is resting on a pillow or cushion.

## Conclusion

The main goal of this paper was to explore the effects of adding background music to an auditory BCI approach that used three stimuli (Halder et al., 2010). Results showed that the users preferred background music to the canonical approach (no background music) without significant changes in BCI performance. While auditory BCIs have been validated in prior work (Hill et al., 2004; Kübler et al., 2009; Treder et al., 2014; Lesenfants et al., 2016; Ortner et al., accepted), this outcome suggests that future auditory, and perhaps other, BCIs could improve user satisfaction by incorporating background music. Further work is needed to explore issues such as: the best signal processing methods and classifiers; performance with target patients at different locations; improving performance with inefficient subjects; and different types of auditory stimuli and background music, including music chosen based on each subject's mental state (Tseng et al., 2015).

## Author contributions

SZ operated the experiment and analyzed the data. BA improved the paper in discussion and introduction. AK improved the experiment and the method and result part. AC offer help in algorithm. JJ offered the idea of this paper and wrote the paper. Dr. Yu zhang help in classification method. XW guided the experiment.

## Funding

This work was supported in part by the Grant National Natural Science Foundation of China, under Grant Nos. 61573142, 61203127, 91420302, and 61305028. This work was also supported by the Fundamental Research Funds for the Central Universities (WG1414005, WH1314023, and WH1516018) and Shanghai Chenguang Program under Grant 14CG31.

### Conflict of interest statement

The authors declare that the research was conducted in the absence of any commercial or financial relationships that could be construed as a potential conflict of interest.
